# Clinico-Etiological Profile of Young-Onset Dementia From a Tertiary Care Center in Northern India

**DOI:** 10.7759/cureus.96203

**Published:** 2025-11-06

**Authors:** Pratyush Kumar, Ashwani Bhat, Deepak Goel, Manish Mittal

**Affiliations:** 1 Department of Neurology, Himalayan Institute of Medical Sciences, Dehradun, IND

**Keywords:** alzheimer's disease, cognitive assessment, dementia vascular, vlom ratio, young-onset dementia

## Abstract

Background: This study examines young-onset dementia (YOD). Young-onset dementia refers to cases of dementia that manifest earlier in life and often pose unique clinical and management challenges. Despite its significant impact, data on YOD in northern India remain limited.

Objective: To evaluate the clinico-etiological profile and cognitive characteristics of young-onset dementia patients presenting to a tertiary care center in Uttarakhand, India.

Methods: This is a longitudinal follow-up study that was conducted over 1.5 years in the Department of Neurology. A total of 37 patients under 65 years of age out of 40 selected, diagnosed with major neurocognitive disorder (DSM-5), were included. Comprehensive clinical assessments, brain imaging, and cognitive evaluations were conducted. Data were analyzed using IBM Corp. Released 2018. IBM SPSS Statistics for Windows, Version 24. Armonk, NY: IBM Corp., with chi-square tests, analysis of variance (ANOVA), and Bonferroni corrections, with significance set at p<0.05.

Results: The majority of patients were males (65%) and aged 56 to 65 years (50%). Vascular dementia was the most common cause (37.8%), followed by Alzheimer’s disease (18.9%) and frontotemporal dementia (13.5%). Based on the verbal-language/orientation-memory (VLOM) ratio, 27.5% had frontotemporal-type dementia and 7.5% had Alzheimer-type dementia. Significant cognitive decline (p < 0.05) in MMSE and ACE-III scores was observed in Alzheimer’s disease and frontotemporal dementia. Vitamin B12 deficiency showed 13.5% improvement with treatment. VLOM ratios effectively differentiated frontotemporal dementia (FTD) from AD.

Conclusion: This study concluded that vascular dementia is the leading cause of YOD in this region, reflecting a high burden of modifiable vascular risk factors. The VLOM ratio offers diagnostic value in distinguishing dementia subtypes. Early identification and intervention are essential to address the functional burden of YOD.

## Introduction

Dementia is a neuropsychiatric disorder that causes gradual cognitive deterioration and dependence on others for basic daily activities (ADL). Dementia usually results in a growing reliance on assistance for daily activities. According to DSM-5 criteria, the term ‘dementia’ has been redefined as ‘major neurocognitive disorder.’ According to the criteria, it causes a decline of memory, attention, or language function, evaluated by clinical examination. The symptoms of the neurodegenerative disorder include the loss of memory, impaired judgment, language issues, disorientation, and some behavioral alterations [1]. A patient's everyday functioning is often evaluated based on their ability to perform instrumental activities of daily living (IADLs), such as managing finances or taking medication. In more severe cases, this includes basic activities of daily living (ADLs), such as feeding or grooming [2].

The risk of dementia is thought to double every five years; it primarily affects those over 65. Young-onset dementia (YOD) refers to dementia symptoms that manifest before the age of 65 [3]. YOD may result from sporadic or inherited etiologies, including neurodegenerative disorders, vascular cognitive impairment, and adult-onset inborn errors of metabolism [4-6]. In addition, it includes various metabolic and storage diseases and other syndromic diagnoses, which are only a few differentials in young-onset dementia. The most common causes of dementia in both older and younger adults (but rarely in those under 35) include Alzheimer's disease, vascular disease, frontotemporal lobar degeneration (FTLD), and dementia with Lewy bodies. However, the clinical presentation of these disorders in younger patients may differ from that typically seen in older adults. The causes of neurodegenerative disorders include Parkinson's disease with dementia, Huntington's disease, Alzheimer's disease, and frontotemporal dementia (FTD) [4].

The study by Harvey et al. and Ikejima et al. reported that YOD values range from 42.3 to 54.0 per 100,000 population [5-7]. According to another study conducted in Norway by Kvello-Alme et al., the incidence rates of dementia were 14.8 and 25.0 per 100,000 person-years for the 30-64 and 45-64 age groups, respectively. Alzheimer's disease was the most common cause. Most patients over 50 had neurodegenerative diseases, whereas younger patients more commonly had non-degenerative disorders. Another study in Italy by Chiari et al. reported an incidence rate of 13.2 per 1,000 person-years (January 2017 to June 2019) [8]. Similarly, research in India has revealed inconsistent patterns in the prevalence and types of young-onset dementia. Diffuse Lewy body disease (DLBD) (2.7%), FTD Parkinson's disease (PD) (2.8%), vascular dementia (5.6%), FTD behavioral variant (23.13%), and Alzheimer's disease (AD) (53.6%) are the most common types, according to Reddy Mukku et al.'s assessment of a 33.7% hospital prevalence. During the four years (January 2004-2008), Nandi et al. discovered that 94/379 (24.55%) of the 379 participants were young adults. Nearly one-third of these incidents involved women. Alzheimer's disease was the most common, accounting for 33% of cases. Huntington's disease (4%) and Parkinson's disease with dementia (4%), vascular dementia (20%), FTD (27%), and miscellaneous (11%) were additional common causes. Degenerative dementia remains the most common cause, even in patients under 65. To date, no studies have specifically addressed early-onset dementia in northern India. We hypothesize that vascular dementia and nutritional causes are the most frequently encountered etiologies in our region, differing from patterns reported elsewhere in the country. The objective of the study is the evaluation of the clinico-etiological profile and also the assessment of the cognitive intelligence among the young group of patients suffering from dementia [9].

## Materials and methods

Research design

This prospective longitudinal observational study was conducted among 37 patients in the Department of Neurology at a tertiary care center in Uttarakhand, Northern India. A purposive, hospital-based convenience sampling technique was used. The study was conducted over one year, from May 20, 2024, to May 15, 2025. It aimed to investigate the epidemiological aspects of neurocognitive disorders in the region. The study received approval from the institutional ethics committee. Patients were enrolled from the Emergency Department, Outpatient Department (OPD), and Specialty Clinics. Inclusion criteria included individuals under 65 years of age diagnosed with major neurocognitive disorder according to DSM-5 criteria who had undergone a comprehensive clinical evaluation and brain imaging. Informed consent was obtained from all participants or their caregivers. A formal power calculation was not performed due to the exploratory nature of this study. The final sample of 37 patients out of a total of 40 represents all eligible cases meeting the inclusion criteria during the study period. Exclusion criteria included patients aged 65 years or older, those unwilling to participate, individuals with space-occupying lesions or lobar hemorrhages, those lacking complete evaluations or imaging, and cases with other causes of altered mental status (e.g., delirium, intoxication). Initially, 40 patients were selected, but three patients lost to follow-up or with inadequate follow-up data were excluded, and 37 patients were selected for the study. Data were collected through structured interviews with patients or caregivers, along with bedside clinical assessments.

Participant recruitment and sampling

Patients were recruited consecutively from the Emergency Department, Outpatient Department, and Specialty Neurology Clinics. A purposive convenience sampling technique was employed, as the study was exploratory in nature and intended to capture all eligible cases within the study period. Initially, 40 patients fulfilled the inclusion criteria; however, three were excluded due to inadequate follow-up data, resulting in a final sample size of 37. No formal a priori power calculation was performed, since the primary aim was descriptive and exploratory rather than hypothesis-driven. Nevertheless, all cases meeting the eligibility criteria during the recruitment period were included, thereby minimizing sampling bias.

Inclusion and exclusion criteria

The inclusion criteria included patients aged below 65 years, diagnosed with major neurocognitive disorder as per DSM-5 criteria, admitted to or attending the emergency, outpatient department (OPD), or specialty clinics of the tertiary care center, patients or caregivers who provided informed consent, and availability of complete clinical evaluation and brain imaging (MRI).

Exclusion criteria included patients aged 65 years or older, patients or caregivers unwilling to participate, patients with space-occupying lesions or lobar hemorrhages on imaging, patients with dementia mimics or other causes of acute altered mental status (e.g., delirium, intoxication), patients who do not have a thorough clinical evaluation or are missing imaging data, and cases with inadequate follow-up data or those lost to follow-up during the study period.

Cognitive assessment

Detailed histories were obtained from all participants. Comprehensive general and systemic examinations were conducted, with particular focus on the neurological system. Additionally, these patients were cognitively screened using the Mini-Mental State Examination (MMSE). The MMSE is a cognitive screening tool that scores up to 30 points, assessing various areas of mental function. It evaluates orientation (10 points), registration (3 points), attention and calculation (5 points), recall (3 points), language (8 points), and visuospatial ability (1 point). The total score can range from 0 to 30, with higher scores indicating better cognitive health. Generally, a score of 24 or above is seen as normal, while scores between 19 and 23 suggest mild cognitive impairment, 10 to 18 indicate moderate impairment, and scores of 9 or below point to severe impairment. The MMSE has been extensively validated for identifying dementia, although it tends to be less sensitive in detecting early-stage dementia and among individuals with lower educational backgrounds. The Hindi version of the MMSE devised by Ganguli et al. in 1995 was employed because the "study population" was the Hindi-speaking belt. The Hindi HMSE comprises 22 items that encompass various aspects of intellectual capability. A cutoff score below 19 indicated dementia, while scores between 19 and 23 suggested mild cognitive impairment. Furthermore, we employed the Addenbrooke's Cognitive Evaluation Hindi version, which is already validated for the Hindi language. Dementia symptoms were measured using the Clinical Dementia Rating Scale. The Clinical Dementia Rating (CDR) Scale evaluates six key areas: memory, orientation, judgment and problem-solving, community affairs, home and hobbies, and personal care. Each of these areas is scored on a 5-point scale (0 = none, 0.5 = very mild, 1 = mild, 2 = moderate, 3 = severe impairment), which helps to create a global CDR score that stages the level of dementia. Here’s how to interpret the scores: 0 means no dementia, 0.5 indicates questionable dementia, 1 is mild dementia, 2 is moderate, and 3 signifies severe dementia. The CDR is known for its strong reliability and validity in distinguishing between different levels of dementia severity and tracking its progression. We opted for a follow-up after three months because this timeframe is crucial for spotting short-term changes in cognition. This is particularly important for conditions that might be reversible, like vitamin B12 deficiency or autoimmune issues, where we expect to see a quick response to treatment. On the flip side, three months is also sufficient to notice significant declines in progressive conditions such as Alzheimer’s disease or vascular dementia. While a longer follow-up could have offered more insights, the one-year study duration and practical limitations meant we couldn't conduct repeated assessments.

All enrolled patients underwent a baseline cognitive and clinical assessment at the time of recruitment. A single follow-up assessment was conducted three months after the initial evaluation to assess changes in cognitive function over time. This follow-up included repeat administration of the MMSE, ACE-III, and VLOM ratio, along with updated clinical evaluations. No additional follow-up time points were included during the one-year study duration. The Verbal-Language/Orientation-Memory (VLOM) ratio was initially measured at baseline (during the presentation) and then again at follow-up, using the subdomain scores from the Addenbrooke’s Cognitive Examination-III (ACE-III). The Hindi version of the Addenbrooke’s Cognitive Examination III (ACE-III) includes 100 items spread across five key areas: attention (18 items), memory (26 items), fluency (14 items), language (26 items), and visuospatial skills (16 items). The highest possible score is 100, with lower scores indicating more significant cognitive impairment. A score of 88 or above is considered normal, while scores between 82 and 87 are seen as borderline, and anything 81 or below suggests cognitive impairment. This Hindi version has been validated for use in Indian populations and shows strong reliability and cultural relevance for screening dementia and making differential diagnoses. 

This ratio is calculated by adding the scores from the verbal fluency and language domains and then dividing that sum by the total of the orientation and memory domain scores. A higher VLOM ratio typically points to a profile that aligns with frontotemporal dementia, while a lower or normal ratio is more indicative of Alzheimer’s disease. Patients who were lost to follow-up or missed the three-month reassessment were excluded from the final analysis.

Brain imaging

Magnetic resonance imaging (3 Tesla) and MR angiography were conducted during recruitment of the participants and included coronal T1-weighted 3D magnetization-prepared fast acquisition with gradient echo, axial fluid-attenuated inversion recovery (FLAIR), axial spin-echo T2-weighted images, and MR angiography. 

Statistical analysis

Data analysis was performed using Microsoft Excel (Redmond, USA) and IBM Corp. Released 2018. IBM SPSS Statistics for Windows, Version 24. Armonk, NY: IBM Corp. Numerical variables are presented as means ± standard deviations, while categorical variables are expressed as counts and percentages. We utilized the chi-square test or Fisher's exact test (as appropriate) to compare dichotomous variables among the various vascular dementia subtypes for risk factors and baseline demographics, while continuous data were analyzed using one-way analysis of variance (ANOVA). The Bonferroni correction was used for numerous correlations. The chi-square test was used to compare the various cognitive domains across the VaD groups. Statistical significance was determined at p < 0.05.

## Results

We conducted our study with 37 patients who met the inclusion criteria. Most of the participants were in the 56-65 age range, which is on the older side of the young-onset dementia spectrum. Interestingly, our cohort had a higher number of males, indicating a male predominance. When we looked at the socio-demographic distribution, we found that a significant number of patients lived in urban areas, which aligns with the hospital’s urban catchment zone. Analyzing their educational backgrounds, we discovered that the largest group had completed high school, suggesting that moderate educational attainment was quite common among them. Regarding the duration of their illness, most patients reported experiencing cognitive decline for about a year before seeking medical help, highlighting that many waited a considerable time before addressing their progressive symptoms.

Table 1 details the distribution of dementia etiologies among the 37 patients studied. The duration of symptoms from the onset to the first hospital visit was one year. Vascular dementia emerged as the most common cause, accounting for 37.8% of cases, with a strong male predominance (85.7%). The majority of patients were males (65%) and aged 56 to 65 years (50%). Vascular dementia was the most common cause (37.8%), followed by Alzheimer’s disease (18.9%) and frontotemporal dementia (13.5%). Alzheimer's disease was the second most frequent etiology (18.9%), also more common in males (71.4%). Fronto-temporal dementia (13.5%) showed a reverse pattern, with higher representation in females (60%). Mild cognitive impairment (MCI), vitamin B12 deficiency, and miscellaneous causes (including Parkinson’s disease and amyloid angiopathy) each accounted for 13.5%, 13.5%, and 8.1%, respectively. Notably, all groups except frontotemporal dementia showed a male majority, suggesting possible gender-based differences in underlying risk factors or diagnostic patterns. The high prevalence of vascular dementia underscores the burden of modifiable cardiovascular risk factors in the population studied.

**Table 1:** Gender-wise distribution of etiologies of dementia MCI: Mild cognitive impairment #: Include amyloid angiopathy, Parkinson’s disease, dementia

Etiology	Male		Female		Total
	Frequency	% out of male	Frequency	% out of female	Frequency
Alzheimer's Disease	5	19.24	2	18.19	7
Fronto-Temporal Dementia	2	7.7	3	27.28	5
Vascular Dementia	10	38.47	2	18.19	12
MCI	3	11.54	2	18.19	5
Vitamin B12 Deficiency	4	15.39	1	9.1	5
Miscellaneous#	2	7.7	1	9.1	3
Total	26	100	11	100	37

Table 2 presents the sex-wise distribution of cognitive patterns among patients with young-onset dementia, assessed using MMSE, ACE III, and VLOM ratio. In our study, most of the patients were from urban areas and were educated up to high school. This could be due to increased awareness about cognitive and behavioral symptoms in these patients. The increased prevalence among men in our study is probably a reflection of societal attitudes, whereby women are often not brought to the hospital in non-emergent conditions. According to MMSE scores, 72.5% of patients had dementia (scores <23), with a male predominance (65.5%), while 20% had mild cognitive impairment (MMSE 23-27). Similarly, 72.5% of patients showed dementia-level impairment on ACE III (<82), with males constituting the majority (62.1%). In the ACE III 82-88 range, all patients were male, while among those with normal scores (≥88), females were more represented (66.7%). VLOM ratio analysis revealed that 27.5% had frontotemporal-type dementia (VLOM <2.2), again more common in males. Interestingly, all patients with Alzheimer-type VLOM ratios (>3.2) were female, suggesting a gender-based pattern in dementia subtypes. The VLOM ratio is clinically relevant, as it classifies different dementias. The low ratio suggests frontotemporal dementia, and the (>3.2) suggests the high ratio of Alzheimer’s disease. This study suggests the abnormality regarding the cognitive domain, which provides accuracy in diagnosis. Overall, most patients exhibited cognitive impairment, with males dominating most categories except for Alzheimer-type dementia, which was exclusively observed in females in this cohort.

**Table 2:** Sex-wise distribution of cognitive patterns in young-onset dementia patients MMSE: Mini Mental State Examination, VLOM: VLOM: Verbal Learning and Organizing Memory Ratio, ACE-III: Addenbrooke's Cognitive Examination III

Cognitive Pattern	Male	Female	Total
	Frequency (%)	Frequency (%)	Frequency (%)
MMSE Score			
<23 (Dementia)	19 (65.5)	10 (35.5)	29 (72.5)
23 – 27 (Mild Cognitive Impairment)	5 (62.5)	3 (37.5)	8 (20)
ACE III			
<82 (Dementia)	18 (62.1)	11 (37.9)	29 (72.5)
82 - 88	5 (100)	0 (0)	5 (12.5)
≥88 (Normal)	1 (33.3)	2 (66.7)	3 (7.5)
VLOM ratio			
<2.2 (Frontotemporal type dementia)	7 (63.6)	4 (36.4)	11 (27.5)
2.2 – 3.2 (Normal)	17 (77.3)	5 (22.7)	22 (55)
>3.2 (Alzheimer-type dementia)	0 (0)	3 (100)	3 (7.5)

Table 3 presents the cognitive patterns of patients at the time of presentation and during follow-up, measured using three cognitive assessment tools: MMSE score, ACE III, and VLOM ratio. The mean MMSE score slightly decreased from 19.6 ± 5.1 to 19.1 ± 5.8, t(36) = -1.68, p = 0.101. Similarly, the ACE III score showed a slight reduction from 65.2 ± 19.8 to 64.9 ± 20.7, with no significant difference (p = 0.321). The VLOM ratio remained virtually unchanged, increasing marginally from 2.4 ± 0.6 to 2.5 ± 0.5, which also lacked statistical significance (p = 0.964). Overall, these results indicate that there was no significant change in cognitive function among the patients between the time of initial presentation and the follow-up assessment.

**Table 3:** Cognitive pattern among patients at the time of presentation and during follow-up MMSE: Mini Mental State Examination, ACE-III: Addenbrooke's Cognitive Examination III, VLOM: Verbal Learning and Organizing Memory Ratio

Cognitive Measure	At Presentation Mean ± SD	At Follow-Up Mean ± SD	t (df)
MMSE Score	19.6 ± 5.1	19.1 ± 5.8	t(36) = -1.68
ACE-III Score	65.2 ± 19.8	64.9 ± 20.7	t(36) = -1.01
VLOM Ratio	2.4 ± 0.6	2.5 ± 0.5	t(36) = 0.05

In Alzheimer's disease, the ACE-III score at presentation was 46.6 ± 18.5, but it slightly decreased to 44.6 ± 18.4 during follow-up. The VLOM ratio remained relatively stable, starting at 3.2 ± 0.3 and maintaining at 3.2 ± 0.2. The MMSE score, however, exhibited a decline from 18.8 ± 1.3 at presentation to 16.8 ± 1.3 at follow-up, suggesting worsening cognitive function over time. For frontotemporal dementia, the VLOM ratio hovered around 1.7, with minimal fluctuation between presentation (1.7 ± 0.1) and follow-up (1.7 ± 0.2), indicating a consistent cognitive pattern across the observation period. Vascular dementia saw a small decline in cognitive scores. The MMSE score dropped from 19.1 ± 4.4 at presentation to 18.3 ± 4.5 during follow-up. Similarly, the ACE-III score also showed a slight decrease from 65.7 ± 19.2 to 63.6 ± 19.0, pointing to a mild cognitive decline over time. In contrast, mild cognitive impairment demonstrated improvement in certain cognitive measures. The MMSE score improved from 25.7 ± 0.9 at presentation to 27.2 ± 0.9 during follow-up. Both the ACE-III score and VLOM ratio also saw slight increases, with ACE-III moving from 87.0 ± 2.4 to 88.2 ± 4.3 and the VLOM ratio rising from 2.7 ± 0.2 to 2.8 ± 0.1. Finally, vitamin B12 deficiency showed a positive trend in cognitive performance. The ACE-III score increased from 79.7 ± 14.7 at presentation to 86.7 ± 3.9 at follow-up, while the VLOM ratio also improved from 2.3 ± 0.5 to 2.6 ± 0.2.

Therefore, Table 4 shows Alzheimer's disease and vascular dementia displayed cognitive decline over time, while mild cognitive impairment and vitamin B12 deficiency had shown improvement or stability, highlighting the varying trajectories of cognitive decline and recovery across different etiologies.

**Table 4:** Cognitive pattern among different etiologies at the time of presentation and during follow-up ACE-III: Addenbrooke's Cognitive Examination III; VLOM: Verbal Learning and Organizing Memory Ratio; MMSE: Mini-Mental State Examination; t-Statistic Value; Probability Value.

Etiology	Measure	At Presentation (Mean ± SD)	At Follow-Up (Mean ± SD)	t-value	P-value
Alzheimer’s Disease	ACE-III	46.6 ± 18.5	44.6 ± 18.4	1.08	0.31
	VLOM Ratio	3.2 ± 0.3	3.2 ± 0.2	1.4	0.58
	MMSE	18.8 ± 1.3	16.8 ± 1.3	4.59	0.001
Fronto-Temporal Dementia	VLOM Ratio	1.7 ± 0.1	1.7 ± 0.2	1.1	0.69
Vascular Dementia	MMSE	19.1 ± 4.4	18.3 ± 4.5	1.8	0.11
	ACE-III	65.7 ± 19.2	63.6 ± 19.0	1.1	0.3
Mild Cognitive Impairment	MMSE	25.7 ± 0.9	27.2 ± 0.9	5	0.0005
	ACE-III	87.0 ± 2.4	88.2 ± 4.3	0.88	0.4
	VLOM Ratio	2.7 ± 0.2	2.8 ± 0.1	1.3	0.85
Vitamin B12 Deficiency	ACE-III	79.7 ± 14.7	86.7 ± 3.9	3.64	0.005
	VLOM Ratio	2.3 ± 0.5	2.6 ± 0.2	1.89	0.08

## Discussion

Our study was conducted among 37 patients and included those participants who were in the age group of 56 to 65 years, and most of the patients were males. In addition, most of the patients were from urban areas and were educated up to high school, and the maximum number of patients had a duration of one year from onset. The most common etiology among the studied patients was vascular dementia, followed by Alzheimer’s disease and frontotemporal dementia. There was a statistically significant difference in the MMSE at the time of presentation and after follow-up of the patients with Alzheimer's disease, vascular dementia, and vitamin B12 deficiency. The AD and FTD cases primarily presented with moderate stages of dementia, while the vascular dementia (VaD) cases were primarily mild to moderate. MCI cases were on the mild side. The differences were statistically significant [10-12].

The prevalence of cognitive impairment is increasing with the growth in life expectancy, as well as the increased incidence of risk factors of dementia. In this context, studies on young-onset dementia are of utmost significance since these patients may present with multiple features that are not seen in late-onset dementia. Though the frequency of YOD is low, it is increasing globally due to the increased incidence of risk factors for dementia. As per the study conducted by Chiari A et al. (2020), 20% of the patients attending the cognition clinic had young-onset dementia. This is in correlation with previous studies from India as well as globally, which have shown a similar frequency of YOD. The Global Burden of Disease data in 2024 estimates the incidence of YOD as 11 per 1,00,000 people, while the prevalence is 119 per 1,00,000 people [13]. The difference in our study could be primarily because this is a hospital-based study and is prone to multiple biases, including referral bias. True prevalence can only be detected in community-based epidemiological studies.

Most of these patients were between the ages of 55 and 65 years. This is in concordance with multiple previous studies [14,15]. The majority of patients were males, and this finding has also been shown in many previous studies [14,16]. In our study, most of the patients were from urban areas and were educated up to high school. This could be due to increased awareness about cognitive and behavioral symptoms in these patients. The increased prevalence among men in our study is probably a reflection of societal attitudes, whereby women are often not brought to the hospital in non-emergent conditions [17]. There could be multiple reasons for the same, including the presence of vascular dementia and other rapidly progressive dementia subgroups in our sample. Frontotemporal dementia patients commonly have an early age of onset and a rapidly progressive course. Also, early-onset AD can present with a rapidly progressive course [18,19].

The most noteworthy finding in our study was the presence of vascular cognitive impairment as the most common form in the studied sample. This could be due to the increased incidence of vascular risk factors such as hypertension, diabetes mellitus, and smoking in our country [20-22]. Thus, prevention of these risk factors, especially hypertension, would be a prudent initiative not only in managing vascular cognitive impairment but also in decelerating their progression during advanced stages, thereby containing the morbidity associated with the disease. This becomes important, especially in our country, where the hypertensive population we see is the tip of the iceberg, and a large chunk of the hypertensive population either doesn't take any treatment or takes the same treatment erratically [23].

There was a statistically significant decline in the MMSE at the time of presentation and after follow-up of the patients with Alzheimer's disease, vascular dementia, and vitamin B12 deficiency. In all these subgroups, the MMSE decreased significantly during the follow-up period. This is based on the normal history of Alzheimer's disease and vascular dementia [19,24]. Among the five cases of vitamin B12 deficiency, there was a statistically significant development in MMSE scores. The ACE scores also showed an improvement in these patients, but the difference was not statistically significant. However, in one case of vitamin B12 deficiency, there was no improvement in the MMSE despite treatment of the underlying deficiency [25]. This might be because the cognitive impairment cases with vitamin B12 deficiency might have already developed irreversible damage. There was a statistically significant difference in the ACE III at the time of presentation and after follow-up of the patients in Alzheimer's disease, frontotemporal dementia, and vascular dementia. This is again by the natural history of frontotemporal dementia, early-onset Alzheimer's disease, and vascular dementia. However, the MSME significantly decreased in the frontotemporal dementia. The reason for this could be that MMSE is not sensitive to detecting frontotemporal dementia compared to other dementias, especially Alzheimer's disease [18,27]. However, ACE III is a sensitive diagnostic tool for detecting other dementias, especially Alzheimer's disease [19,29]. ACE III was also proven to be a reliable mental screening tool in Alzheimer's disease [30].

We only had six cases of reversible dementia in our study group. Identifying reversible causes of cognitive impairment, like autoimmune encephalitis and vitamin B12 deficiency, is incredibly important in clinical practice. Unlike progressive neurodegenerative dementias, a timely diagnosis and targeted treatment in these situations can actually stop or even reverse cognitive decline, which helps lessen long-term disability and dependence. While our study only looked at six cases, the improvements we saw in cognitive scores after intervention highlight the need for regular screening for reversible causes in all patients who show signs of young-onset dementia. Catching these issues early not only leads to better outcomes for patients but also eases the strain on caregivers and healthcare systems. Five patients had vitamin B12 deficiency. Although there was development in the overall mental status of the case of autoimmune encephalitis, this couldn't be proven objectively, as MMSE and ACE couldn't be applied at the initial presentation of the patient. In all five cases of MCI, the MMSE showed improvement on follow-up, while there was a slight improvement in the ACE-3 scores. However, the difference in either of the cases was not significant.

In the study, the VLOM ratio remained stable for the frontotemporal dementia, with no statistical change in the baseline and follow-up. Based on the VLOM ratio, 11 (27.5%) patients had frontotemporal-type dementia, and three (7.5%) patients had Alzheimer-type dementia. A total of 22 (65%) patients had VLOM in the range of 2.2 to 3.2.

The calculation of the verbal-language/orientation-memory (VLOM) ratio indicates that orientation, attention, and memory are more severely compromised in individuals with Alzheimer's disease (AD), while language functions exhibit more impairment in patients with frontotemporal dementia (FTD) [30]. Previous studies have shown that the sensitivity of the VLOM ratio in diagnosing frontotemporal dementia is low, but its specificity is high. In contrast, the sensitivity in diagnosing Alzheimer’s disease is 72%, and the sensitivity of this condition is 69.4% [27]. Also, it has been shown that the subtests of the ACE-III have significant associations with neuropsychological tests specific to that domain [30]. The VLOM ratio is a powerful neuropsychological tool in differentiating between AD and FTD. Our investigation found a statistically significant connection between the VLOM ratio and particular subtypes of dementia.

Patients with frontotemporal dementia showed different VLOM ratios. This correlates with the pathophysiology of frontotemporal dementia, which affects their frontal and anterior temporal lobes. These are responsible for language, semantic processing, and executive function. However, the episodic memory and orientation deficit are not present in the early stages of FTD. This means that the primary domain that is affected in these patients is executive function and language, and thus, the VLOM ratio is skewed towards global and linguistic dysfunction compared to memory or orientation dysfunction [19].

On the other hand, the VLOM ratio was normal to high in Alzheimer's disease patients. In Alzheimer’s disease, there is early involvement of the medial temporal lobes and hippocampus in the classical variant. This leads to early episodic memory and orientation deficits while their language and fluency are spared in the earlier stages. Thus, the normal or increased VLOM ratio in Alzheimer’s disease suggests a pattern where memory and orientation are disproportionately affected compared to language.

The vascular cognitive impairment group showed a mixed pattern and had both normal and low VLOM ratios. This most likely reflects the underlying heterogeneity that is present in vascular cognitive impairment, as it can involve diffuse cortical as well as cortical structures depending on the location and extent of infarcts. As a result, both executive linguistic defects and memory and orientation deficits can be seen in these patients [29]. Although there were certain limitations, like small sample size and typical or mixed presentations in our samples, we still suggest that VLOM is a reliable cognitive biomarker that helps distinguish between Alzheimer's disease and dementia of the frontal lobe, and it can enhance the clinical diagnostic precision in these cases. The findings are in accordance with the proposed hypothesis, as the VLOM ratio is different for both Alzheimer’s disease and frontotemporal dementia. But no significant changes have been obtained for the FTD, thus providing its limitation for the tracking of the progression of the disease. This is one of the very few studies on young-onset dementia that has been conducted in India, and the first study from our region and the state of Uttarakhand. Another important strength is that a wide variety of etiologies were taken into account while conducting the study. This is one of the very few studies where the volume ratio has been calculated in young-onset dementia patients. The study's limited sample size was its main drawback. The current study does have a few limitations worth mentioning. To start, the sample size was on the smaller side, which could affect how broadly we can apply the findings. Additionally, since this was a hospital-based study, we can't completely rule out referral and selection biases, which make it tricky to accurately gauge the true prevalence of young-onset dementia in the wider community. Another point is that we didn't use functional neuroimaging techniques like positron emission tomography (PET) or single-photon emission computed tomography (SPECT), which could have offered more precise diagnostic insights and helped differentiate between subtypes. Moreover, the presence of atypical or mixed cognitive decline presentations, including overlapping symptoms of Alzheimer’s and other dementias, might have impacted how we interpreted the VLOM ratio. Lastly, the relatively short follow-up period limited our ability to track long-term cognitive changes in these patients. The absence of functional neuroimaging investigations to support our diagnosis was another significant drawback. The VLOM ratio results in our patients may have been impacted by the atypical or mixed presentations of cognitive decline and Alzheimer's disease [30]. Our research has some challenges posed by young-onset dementia, with vascular cognitive impairment being the leading cause. This underscores the importance of managing risk factors early on. To enhance diagnostic accuracy and develop effective preventive strategies, we need larger community-based studies that involve longer follow-up periods and advanced imaging techniques.

## Conclusions

The study has concluded that vascular dementia is the most common cause of young-onset dementia, with a male predominance. Cognitive decline was observed in most patients, but Alzheimer's disease, in particular, showed a decline exclusive to females. Mild cognitive impairment and vitamin B12 deficiency demonstrated stability or improvement. These findings highlight the varying trajectories of dementia based on etiology and gender. The maximum common etiology of YOD in our region is vascular dementia, followed by Alzheimer’s disease and frontotemporal dementia. VLOM ratios are effective in diagnosing frontotemporal dementia.
